# First in human dose calculation of a single-chain bispecific antibody targeting glioma using the MABEL approach

**DOI:** 10.1136/jitc-2019-000213

**Published:** 2020-04-08

**Authors:** Teilo H Schaller, David J Snyder, Ivan Spasojevic, Patrick C Gedeon, Luis Sanchez-Perez, John H Sampson

**Affiliations:** 1 Preston Robert Tisch Brain Tumor Center, Duke University Medical Center, Durham, North Carolina, United States; 2 Department of Neurosurgery, Duke University Medical Center, Durham, North Carolina, United States; 3 Department of Pathology, Duke University Medical Center, Durham, North Carolina, United States; 4 PK/PD Core Laboratory, Duke Cancer Institute, Durham, North Carolina, United States; 5 Department of Medicine, Duke University School of Medicine, Durham, North Carolina, United States

**Keywords:** oncology, neurooncology, immunology, pharmacokinetics

## Abstract

**Background:**

First-in-human (FIH) clinical trials require careful selection of a safe yet biologically relevant starting dose. Typically, such starting doses are selected based on toxicity studies in a pharmacologically relevant animal model. However, with the advent of target-specific and highly active immunotherapeutics, both the Food and Drug Administration and the European Medicines Agency have provided guidance that recommend determining a safe starting dose based on a minimum anticipated biological effect level (MABEL) approach.

**Methods:**

We recently developed a T cell activating bispecific antibody that effectively treats orthotopic patient-derived malignant glioma and syngeneic glioblastoma in mice (hEGFRvIII:CD3 bi-scFv). hEGFRvIII:CD3 bi-scFv is comprized of two single chain antibody fragments (bi-scFvs) that bind mutant epidermal growth factor receptor variant III (EGFRvIII), a mutation frequently seen in malignant glioma, and human CD3ε on T cells, respectively. In order to establish a FIH dose, we used a MABEL approach to select a safe starting dose for hEGFRvIII:CD3 bi-scFv, based on a combination of in vitro data, in vivo animal studies, and theoretical human receptor occupancy modeling.

**Results:**

Using the most conservative approach to the MABEL assessment, a dose of 57.4 ng hEGFRvIII:CD3 bi-scFv/kg body weight was selected as a safe starting dose for a FIH clinical study.

**Conclusions:**

The comparison of our MABEL-based starting dose to our in vivo efficacious dose and the theoretical human receptor occupancy strongly supports that our human starting dose of 57.4 ng hEGFRvIII:CD3 bi-scFv/patient kg will be safe.

## Background

The past decade has seen multiple FDA approvals of tumor-specific immune system-activating therapies, generally referred to as cancer immunotherapy.^1 2^ These therapies range from immune modulators targeting programmed cell death protein 1 (PD-1), programmed death-ligant 1 (PD-L1), or cytotoxic T-lymphocyte-associated protein 4 (CTLA-4) to cellular therapies using dendritic cells or genetically engineered T cells and monospecific or bispecific antibodies targeting tumor antigens. Immunotherapies have shown unprecedented improvements in survival in numerous hematologic and solid tumors, providing hope to patients who have exhausted traditional treatment regimens.^3 4^

Malignant gliomas are the most common primary brain tumors in adults. Glioblastoma (GBM), a grade 4 glioma, makes up >50% of malignant gliomas and has an incidence rate of 3.2 per 100,000 population. Median survival of patients with GBM is around ~20 months, despite aggressive image-guided tumor resection, external-beam radiation, chemotherapy, such as temozolomide, and tumor-treating fields.^5^ While there are currently no immunotherapies available for GBM, we and others are developing various tumor-specific treatments.^6–9^ Recently, using a combination of two single-chain variable fragments (scFv) with different specificities, we developed a novel bispecific antibody to treat GBM. Our antibody targets with one arm the human CD3ε receptor on T cells and the tumor-specific epidermal growth factor receptor variant III (EGFRvIII) mutation with the other arm. Dual binding of this hEGFRvIII:CD3 bi-scFv redirects T cells to lyse GBM cells, independent of T cell receptor specificity.^6^

hEGFRvIII:CD3 bi-scFv is a potent 50.9 kDa antibody that binds specifically to EGFRvIII, but not to EGFR, with an equilibrium constant of dissociation (K_D_) of 27.8 nM.^6^ It also binds specifically to human CD3ε with a K_D_ of 15.6 nM, but not to CD3ε of other species.^6^ hEGFRvIII:CD3 bi-scFv has a short pharmacokinetic profile typical of truncated antibodies. A pharmacokinetic study in human CD3 transgenic mice^10^ revealed that hEGFRvIII:CD3 bi-scFv in plasma and whole blood has an initial half-life of ~8 min and terminal half-life of ~2.5 hours.^11^ Our previous work showed that hEGFRvIII:CD3 bi-scFv induces robust antibody-induced T cell activation, secretion of proinflammatory cytokines, and T cell proliferation only in the presence of target antigen.^6^ The antibody mediates potent tumor cell lysis in multiple human glioma lines and patient-derived glioma samples.^6^ In mice, intravenous hEGFRvIII:CD3 bi-scFv administration effectively treats and extends survival in well-engrafted subcutaneous and orthotopic models of glioma.^6^ We showed extended survival and long-term cures in both patient-derived gliomas using NOD-*scid* gamma (NSG) mice (xenografts) and in highly invasive murine gliomas using transgenic mice engineered to express the human CD3 receptor (syngeneic).^6^

The translation of a novel therapeutic into human clinical studies is regulated by the Food and Drug Administration (FDA). Approval to conduct a first-in-human (FIH) study is based on submission of an investigational new drug (IND) application, which contains extensive information on topics such as in-depth characterization of the drug, proof of preclinical efficacy, toxicology studies, and development of a current Good Manufacturing Practice (cGMP)-regulated manufacturing process. A critical aspect of submitting an IND application to the FDA is the establishment of an appropriate FIH dose. This dose represents the starting point for clinical trials and thus must be absolutely safe, yet also be close to a value expected to have biological activity. Traditionally, the maximum safe starting dose in initial clinical trials for therapeutics is determined based on a toxicology study in a pharmacologically relevant species. As described in a guidance document by the FDA, this process is based on conducting toxicity studies in multiple pharmacologically relevant species to ascertain the maximum dose that does not result in any adverse effects, called the ‘no observed adverse effects levels’ (NOAEL).^12^ After selecting the most appropriate species, based on a multifactorial analysis of sensitivity to the drug, relevance of the species for the targeted mechanism of action, and the applicability of toxicities to humans, the NOAEL is converted to a human equivalent dose (HED). Finally, a safety factor of at least 10 is applied to the dose to obtain the human maximum recommended safe starting dose (MRSD).

However, given the advent of highly active biotherapeutics that can induce serious toxicities including cytokine release syndrome and neurotoxicity at low doses, there is a move towards using first-in-human doses based on anticipated biological effects and not adverse effects.^13 14^ The approach, called minimum anticipated biological effect level (MABEL), first recommended by the European Medicines Agency (EMA) in 2007 and now also recommended by the FDA for certain therapeutics, including bispecific antibodies, is increasingly being used to determine the MRSD in both USA and European trials.^15–17^ In fact, a recent review by Suh *et al* reports a fivefold increase in the use of MABEL for calculating the MRSD of monoclonal antibody trials for the years 2011–2013 compared with the years 1990–2007.^18^ According to guidance published by the EMA:

The calculation of MABEL should utilise all in vitro and in vivo information available from pharmacokinetic/pharmacodynamic (PK/PD) data such as: i) target binding and receptor occupancy studies in vitro in target cells from human and the relevant animal species; ii) concentration-response curves in vitro in target cells from human and the relevant animal species and dose/exposure-response in vivo in the relevant animal species. iii) exposures at pharmacological doses in the relevant animal species. Wherever possible, the above data should be integrated in a PK/PD modelling approach for the determination of the MABEL.^17^

Here we detail our MABEL approach for calculating the MRSD dose of hEGFRvIII:CD3 bi-scFv for a first-in-human clinical trial. We show that cGMP-representative hEGFRvIII:CD3 bi-scFv only binds human CD3ε, thus limiting our preclinical testing to in vitro assays and humanized mouse models. We then determined the most sensitive in vitro pharmacologically relevant assay to calculate a FIH dose using the MABEL approach. This dose was compared with the effective in vivo murine dose and the theoretical receptor occupancy of CD3 in humans to ensure our MABEL approach was both a relevant and a safe dose.

## Materials and methods

### Production of hEGFRvIII:CD3 bi-scFv

hEGFRvIII:CD3 bi-scFv (50.9 kDa) was produced in a similar manner as previously published.^6 11^ The most significant change arises from the outsourcing of the production to a clinical research organization to produce cGMP and cGMP-representative hEGFRvIII:CD3 bi-scFv. Briefly, stably transduced Chinese hamster ovary (CHO-S) cells, thawed from a cryopreserved cGMP master cell bank vial, were expanded in shaker flasks until of sufficient density to start the production run. Production of hEGFRvIII:CD3 bi-scFv occurred over 12 days, during which time the CHO-S cells were regularly fed to ensure high viability. After cell clarifications, hEGFRvIII:CD3 bi-scFv was harvested from the supernatant using affinity chromatography, specifically using MabSelect Protein A, Sartobind Q, and SP-Sepharose columns. Virus removal was done via a low pH hold and 20 nm ultrafiltration using a Planova BioEX filtration system. Purified antibody was stored in sterile vials at a maximum concentration of 0.5 mg/mL. All experiments in this work were done with cGMP-representative material to ensure the most accurate and clinically relevant dosing calculations.

### Binding of hEGFRvIII:CD3 bi-scFv to blood cells

A flow cytometry assay was utilized to confirm binding of hEGFRvIII:CD3 bi-scFv to CD3-expressing blood cells and to establish species specificity of the antibody. Healthy peripheral blood mononuclear cell (PBMC) samples for the species beagle, human, CD-1 mouse, cynomolgus, Sprague Dawley rat, New Zealand white rabbit, and pig were obtained frozen from BioIVT (New York, USA). Human PBMC samples were also obtained from healthy donors at Duke University. The binding assay consisted of a two-step binding protocol that ensured both arms of the bispecific antibody were functional at the same time. Specifically, 1 µg of hEGFRvIII:CD3 bi-scFv was incubated with 1×10^6^ to 3×10^6^ PBMCs for 30 min, which results in binding of CD3ε. The samples were washed and then incubated for 30 min with a custom-made fluorescent EGFRvIII petide (PEPvIII)/Streptavidin-PE multimer (PEPvIII multimer), which binds the other arm of the bispecific antibody. After another wash step, the cells were analyzed in a BD Fortessa flow cytometer. Fluorescence data were analyzed using Flowjo. A fluorescent signal indicates that hEGFRvIII:CD3 bi-scFv binds to CD3-expressing cells of that species and that both arms of the antibody are functional.

### Binding of hEGFRvIII:CD3 bi-scFv to tumor cells

Flow cytometry was used to confirm binding of hEGFRvIII:CD3 bi-scFv to EGFRvIII expressing tumor cells. Cell lines used included the human malignant glioma line U87-MG, both wild type and engineered to express EGFRvIII (U87-MG-EGFRvIII). The binding assay consisted of three steps. In step 1, around 200,000 target tumor cells were incubated with hEGFRvIII:CD3 bi-scFv for 30 min at 4°C. Following that, cells were washed two times in fluorescence-activated cell sorting (FACS) buffer and incubated with Protein-L-biotin (Genscript M00097) for 30 min at 4°C. Finally, after two more washes, cells were incubated with Streptavidin-PE (Biolegend 405204) for 30 min at 4°C. Cells were analyzed in a BD Fortessa flow cytometer. Fluorescence data were analyzed using Flowjo.

### Cytokine release assay

We assessed IFN-ɣ, IL-6, IL-1β, MIP-1α, granulocyte-macrophage colony-stimulating factor (GM-CSF), and tumor necrosis factor (TNFα) release using the Luminex multiplex platform (Millipore HSTCMAG-28SK). Target tumor cells were mixed with human PBMCs at a ratio of 1:20 in a 96-well ultra-low adherent U bottom plate. Human PBMCs alone served as control. hEGFRvIII:CD3 bi-scFv was added to the cells at varying concentrations and the cells were incubated at 37°C. Maximal cytokine release was induced using 50 nM phorbol 12-myristate 13-acetate (PMA). After 24 hours, the plate was spun and supernatant was harvested. Cytokines in the supernatant were measured by the Duke core laboratory RBL Immunology using the Luminex kit.

### T cell activation and proliferation

We assessed hEGFRvIII:CD3 bi-scFv-induced T cell activation by measuring CD25 upregulation and proliferation by measuring 5(6)-carboxyfluorescein (CFSE) dilution. Human PBMCs were labeled with 1 µM CFSE (Invitrogen C34554) for 15 min at 37°C, according to the manufacturer’s recommendations. Target tumor cells were mixed with human PBMCs at a ratio of 1:10 in 96-well U bottom plates. After the addition of hEGFRvIII:CD3 bi-scFv, the cells were placed in an incubator for 5 days at 37°C. Subsequently, the plates were spun, the supernatant was discarded, and the cells were stained with anti-CD3-APC (Biolegend 300412) and anti-CD25 antibody-BV421 (Biolegend 302630). CD25 and CFSE signal was measured using a BD Fortessa flow cytometer.

### Tumor cell lysis

We assessed in vitro target cell cytotoxicity using chromium release assays. ^51^Cr (200 mCi; Perkin Elmer) was used to label 4×10^6^ target cells. Target cells were incubated with 200 µCi of radioactive ^51^Cr for 1 hour at 37°C, and the reaction mixture was agitated every 15 min. Labeled cells were washed three times with phosphate-buffered saline (PBS), resuspended in cell culture media, and allowed to rest for 20 min at room temperature. Target cells were washed an additional time to remove free ^51^Cr and then incubated with various combinations of effector PBMCs and/or bi-scFvs at various concentrations. Effector cells were plated at an E:T ratio of 20:1 when present. Maximal lysis was induced using a 2.0% solution (v/v) of Triton X-100 (Sigma Aldrich). Cells were incubated at 37°C for 20 hours after which 50 µL of supernatant from each well was collected and combined with 150 µL of OptiPhase Supermix Scintilation Cocktail (Perkin Elmer). Radioactivity released into the supernatant was measured using a 1450 MicroBeta TriLux Microplate Scintillation and Luminescence Counter (Perkin Elmer).

### Dose selection based on an in vitro MABEL approach

To obtain a MRSD for our first-in-human study of hEGFRvIII:CD3 bi-scFv, we ascertained the minimum active biological effect level based off of a battery of in vitro assays, the pharmacologically active dose in vivo, and the theoretical receptor occupancy of CD3 in humans.

For all our in vitro assays, we calculated the concentration of hEGFRvIII:CD3 bi-scFv that results in 20% of the maximal effective concentration (EC_20_). The EC_20_ value is a conservative measurement of biological activity and can be converted into a HED by equating EC_20_ to the maximum plasma concentration (C_max_) in humans. Thus, the human equivalent starting dose was calculated as follows, based on an average human blood plasma volume of 3 L:

$$Human\;equivalent\;dose=EC_{20}\;from\;in\;vitro\;assay\;\times \;3\;L\;plasma\;volume$$

The FIH starting dose equals the HED from the in vitro assay with the lowest EC_20_. To place the FIH dose into context, we then compared it to the pharmacologically active dose from our murine studies and to the theoretical occupancy of CD3 receptors in humans.

### In vivo efficacy experiments

All animal experiments were performed under the protocol A224-18-09 approved by the Duke University Institutional Animal Care and Use Committee. Studies were performed as previously described.^6^ Briefly, for orthotopic U87-MG-EGFRvIII glioma models, tumor cells were grown and collected in logarithmic growth phase, washed with PBS, and mixed with an equal volume of 10% methyl cellulose. Cell mixtures were loaded into a 250 µL Hamilton syringe and a 25-gage was attached. Using a stereotactic frame, 5 µL of the cell mixture was injected into the right hemisphere—2 mm to the right of the bregma and 4 mm below the surface of the skull at the caudate nucleus—of anesthetized 8 to 12-week-old female mice. A total of 5×10^4^ U87-MG-EGFRvIII cells mixed with 5×10^4^ human PBMCs were implanted into NSG mice (Jackson Laboratory), with 10 mice per group. In the treatment group, 50 µg hEGFRvIII:CD3 bi-scFv in PBS was given via daily intravenous tail vein injection during the first 5 days after tumor implantation. Mice were euthanized at humane endpoints and survival was recorded.

## Results

### hEGFRvIII:CD3 bi-scFv binds only to human CD3-expressing cells

**Figure 1:**
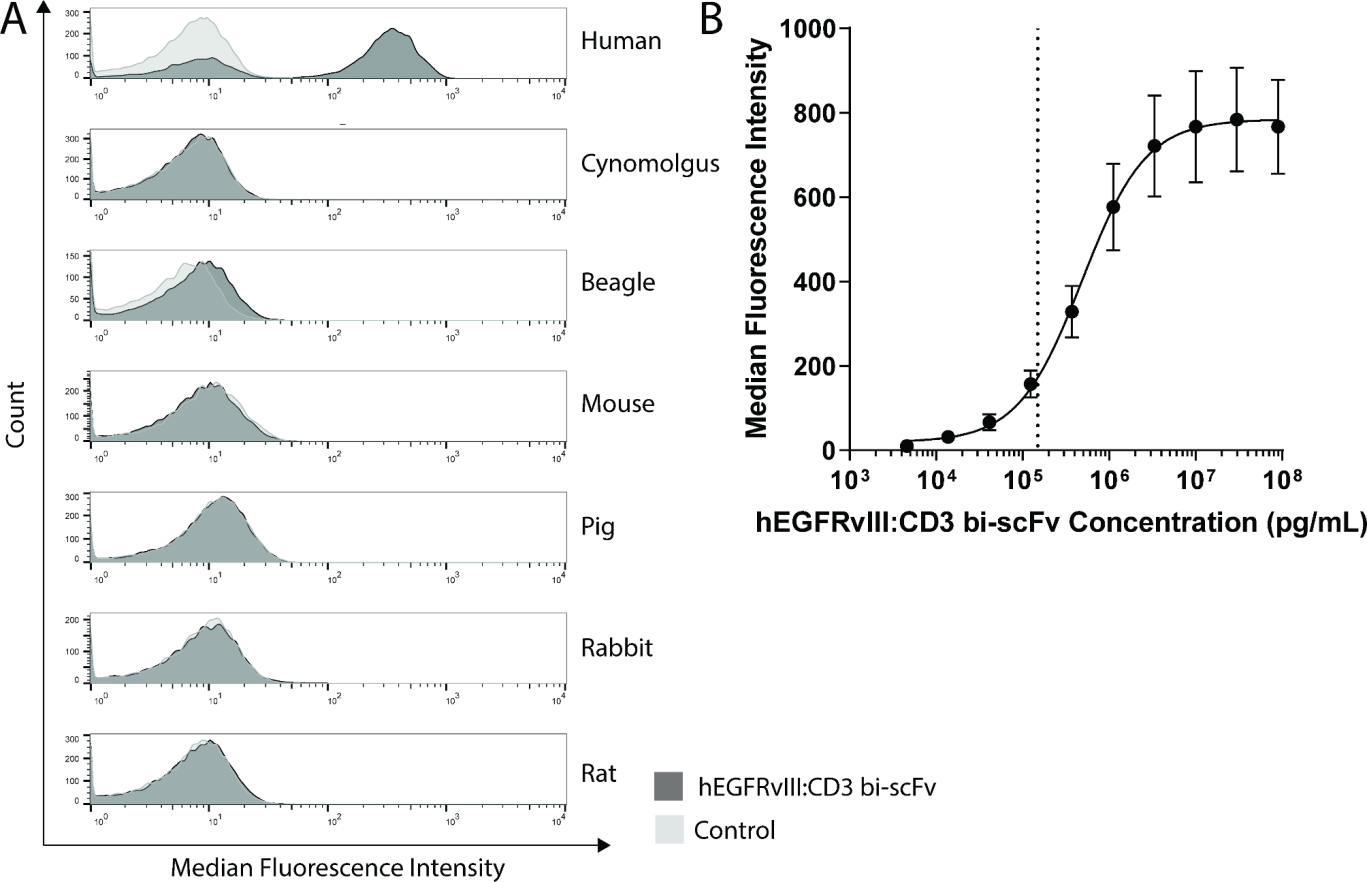
hEGFRvIII:CD3 bi-scFv binds only human CD3-expressing cells. (A) hEGFRvIII:CD3 bi-scFv binding to PBMCs from human, cynomolgus, beagle, mouse, pig, rabbit, and rat were tested using flow cytometry. Binding studies using only the secondary detection PEPvIII multimer served as control. (B) hEGFRvIII:CD3 bi-scFv binding to PBMCs from three human donors. Dashed line shows EC_20_. EC_20_, 20% of the maximal effective concentration; EGFRvIII, epidermal growth factor receptor variant III; PBMCs, peripheral blood mononuclear cells.

Flow cytometry was used to test the binding characteristics of hEGFRvIII:CD3 bi-scFv to PBMCs from various species (figure 1A) and from three human donors (figure 1B). Since hEGFRvIII:CD3 bi-scFv was expressed without purification tags and there are no known antibodies to the drug, we developed a PEPvIII-Biotin/SA-PE multimer to detect hEGFRvIII:CD3 bi-scFv on the surface of cells. The multimer was constructed with the EGFRvIII antigenic determining peptide, PEPvIII, allowing for simultaneous detection of hEGFRvIII:CD3 bi-scFv binding to the surface of lymphocytes and tumor antigen. Of the seven species tested, hEGFRvIII:CD3 bi-scFv bound only to human PBMCs. The multidonor binding assay showed that hEGFRvIII:CD3 bi-scFv binds human PBMCs across a wide concentration range (figure 1B). The CD3 receptors were saturated at and above ~1×10^7^ pg/mL hEGFRvIII:CD3 bi-scFv and the EC_20_ is 150 ng/mL.

### hEGFRvIII:CD3 bi-scFv binds to EGFRvIII-expressing glioma cells

**Figure 2:**
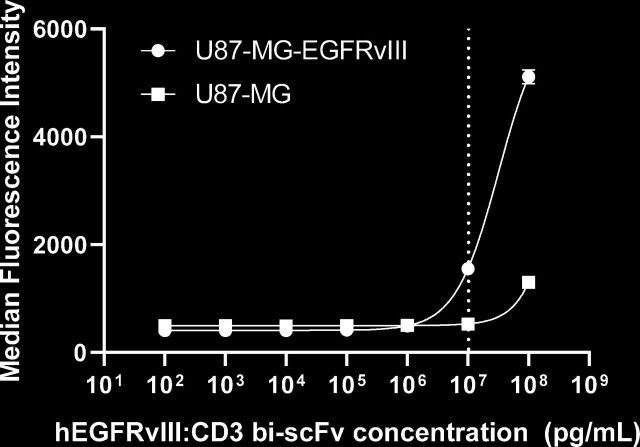
hEGFRvIII:CD3 bi-scFv binds to EGFRvIII. U87-MG-EGFRvIII and control U87-MG cells were incubated first with hEGFRvIII:CD3 bi-scFv and then with fluorescent Protein-L. Fluorescence was measured using a flow cytometer. The dashed line shows the EC_20_ value for binding to U87-MG-EGFRvIII. The line of best fit for U87-MG was ambiguous. EC_20_, 20% of the maximal effective concentration; EGFRvIII, epidermal growth factor receptor variant III.

To assess and confirm functional binding of hEGFRvIII:CD3 bi-scFv to EGFRvIII, we labeled U87-MG-EGFRvIII and U87-MG control cells with our antibody and secondary fluorescent Protein-L. Protein-L binds variable fragments of antibodies and can be used to detect unlabeled molecules. Using flow cytometry, we determined that hEGFRvIII:CD3 bi-scFv binding to EGFRvIII was orders of magnitude more specific than binding to wild type EGFR (figure 2). The EC_20_ for hEGFRvIII:CD3 bi-scFv binding U87-MG-EGFRvIII is 10,307 ng/mL. A line of best fit, and thus the EC_20_, for the U87-MG group could not be accurately calculated.

### hEGFRvIII:CD3 bi-scFv induces cytokine release from CD3-expressing cells in a dose-dependent and antigen-dependent manner

**Figure 3:**
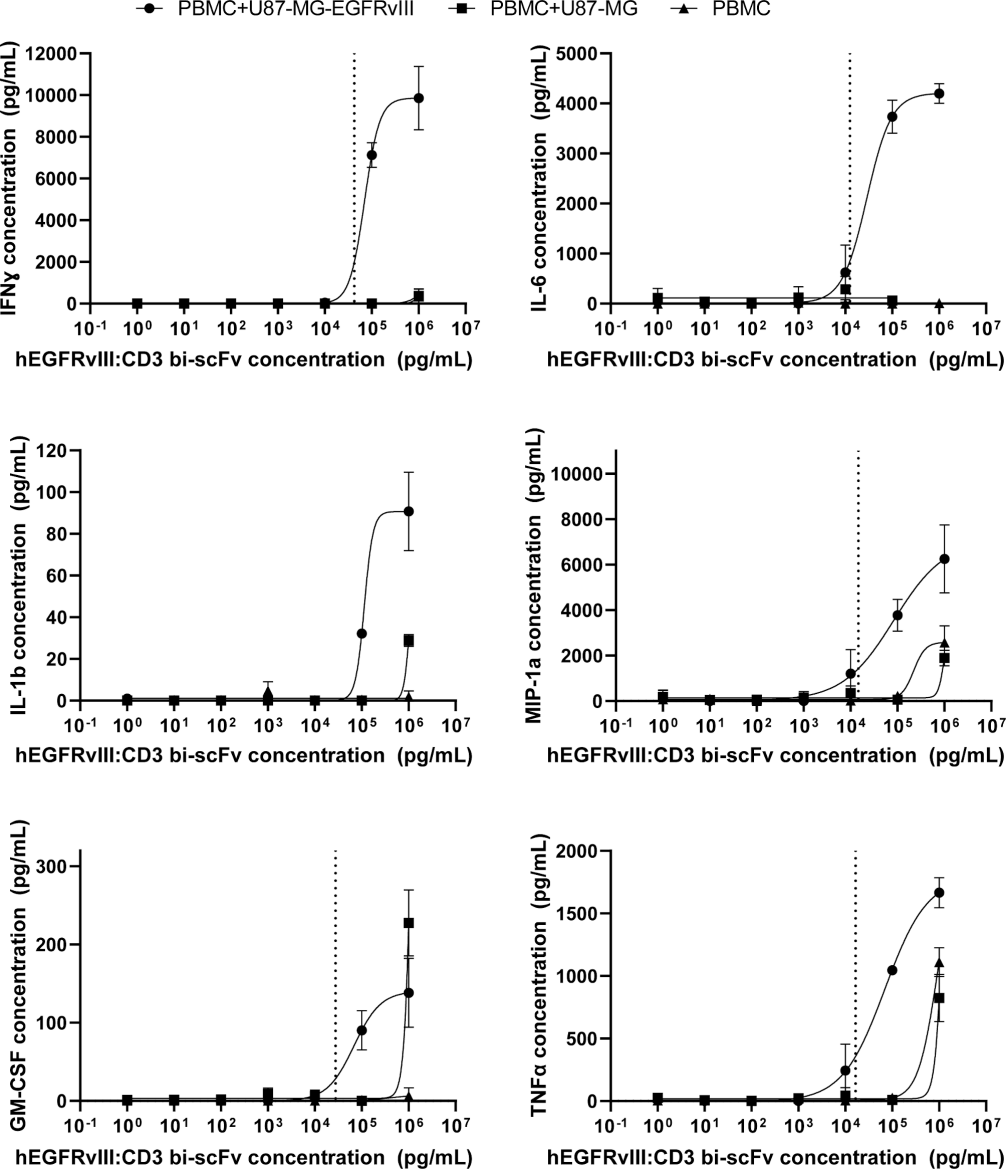
hEGFRvIII:CD3 bi-scFv induces cytokine release from CD3-expressing cells in the presence of target tumor antigen. Using the Luminex multiplex assay, we determined the concentrations of IFN-ɣ, IL-6, IL-1β, MIP-1α, GM-CSF, and TNFα after 24 hours incubation with either U87-MG-EGFRvIII cells, control U87-MG cells, or PBMCs alone. For all cytokines, hEGFRvIII:CD3 bi-scFv significantly activated T cells only in the presence of the target EGFRvIII antigen. Dashed lines represent EC_20_ values for non-ambiguous regressions. U87vIII is U87-MG-EGFRvIII. EC_20_, 20% of the maximal effective concentration; EGFRvIII, epidermal growth factor receptor variant III; PBMCs, peripheral blood mononuclear cells.

We assessed cytokine release to determine the ability of hEGFRvIII:CD3 bi-scFv to induce target antigen-dependent activation of T cells. Following a 24 hours incubation of hEGFRvIII:CD3 bi-scFv, PBMCs, and human glioma cells U87-MG-EGFRvIII or control U87-MG cells, we harvested the supernatant and measured the concentration of IFN-ɣ, IL-6, IL-1β, MIP-1α, GM-CSF, and TNFα using the Luminex multiplex platform (figure 3). A dose relationship between the amount of hEGFRvIII:CD3 bi-scFv and amount of cytokines released from PBMCs was detected. We determined that for all cytokines, hEGFRvIII:CD3 bi-scFv-induced cytokine release was order of magnitudes higher in the presence of U87-MG-EGFRvIII cells than compared with the control U87-MG cells. In fact, for four of the six cytokines tested, hEGFRvIII:CD3 bi-scFv did not elicit increase cytokine release from the control U87-MG cells above that of background. These data show the specificity of hEGFRvIII:CD3 bi-scFv for its target antigen EGFRvIII. Lines of best fit could be accurately calculated for five out of six cytokines in the experimental PBMC +U87-MG-EGFRvIII group but for none of the cytokines in the two control groups (PBMC +U87 MG and PBMC alone). In the experimental group, the cytokine release EC_20_ concentrations hEGFRvIII:CD3 bi-scFv for IFN-ɣ, IL-6, MIP-1α, GM-CSF, and TNFα were 42.3, 12.5, 14.7, 27.6, and 16.5 ng/mL, respectively.

### hEGFRvIII:CD3 bi-scFv activates and induces proliferation of T cells

**Figure 4:**
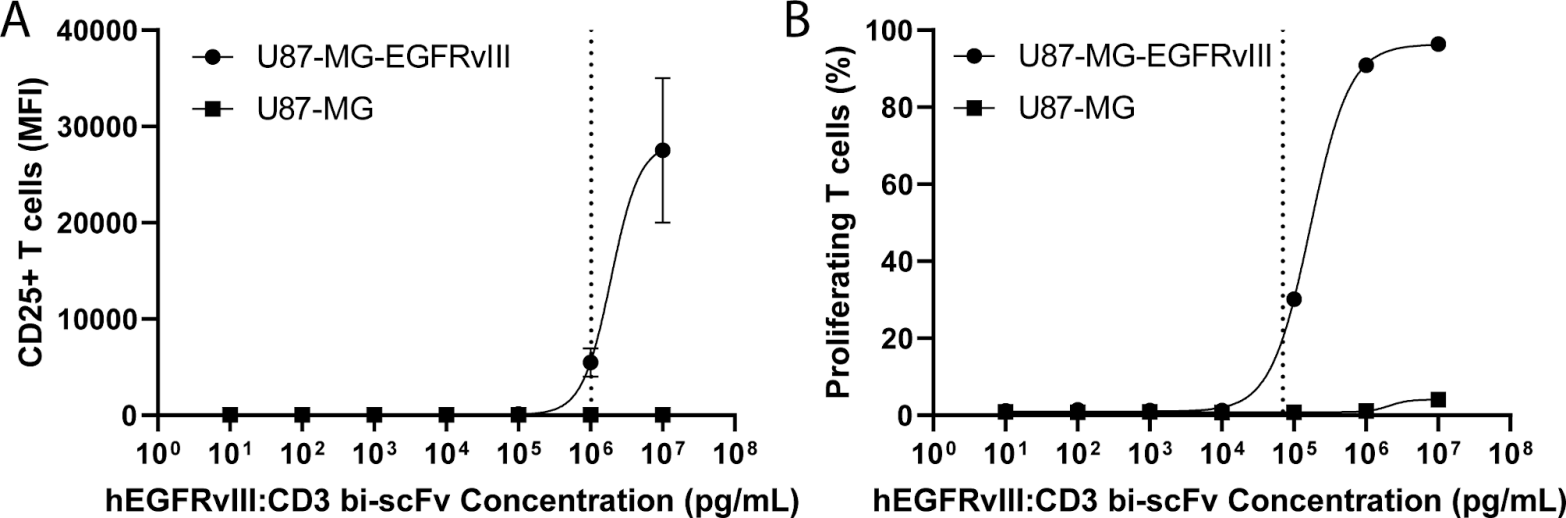
hEGFRvIII:CD3 bi-scFv activates and induces proliferation of T cells. After 5 days of coincubating hEGFRvIII:CD3 bi-scFv, human PBMCs, and U87-MG-EGFRvIII or U87-MG control cells, T cell activation (A) and proliferation (B) were measured. Dashed lines show EC_20_ for U87-MG-EGFRvIII groups. EC_20_, 20% of the maximal effective concentration; EGFRvIII, epidermal growth factor receptor variant III.

We assessed the ability of hEGFRvIII:CD3 bi-scFv to induce T cell activation and proliferation by coincubating hEGFRvIII:CD3 bi-scFv, human PBMCs, and U87-MG-EGFRvIII or U87-MG cells. After 5 days, T cell activation was analyzed by measuring CD25 expression and T cell proliferation was assessed by measuring CFSE incorporation. hEGFRvIII:CD3 bi-scFv activated and induced proliferation of T cells in a target antigen-specific and dose-dependent manner (figure 4). The EC_20_ concentration of hEGFRvIII:CD3 bi-scFv for T cell activation and proliferation is 1021 and 70.2 ng/mL, respectively. No lines of best fit could be accurately calculated for the U87-MG control group.

### hEGFRvIII:CD3 bi-scFv-induced tumor cell lysis

We previously showed that hEGFRvIII:CD3 bi-scFv induces tumor cell lysis in a dose-specific and antigen-specific manner.^6^ In those studies, chromium cytotoxicity assays were performed with two glioma cell lines engineered to express EGFRvIII, U87-MG-EGFRvIII and D54-EGFRvIII, and with a patient-derived cell line that naturally expresses EGFRvIII, D270-MG.

**Figure 5:**
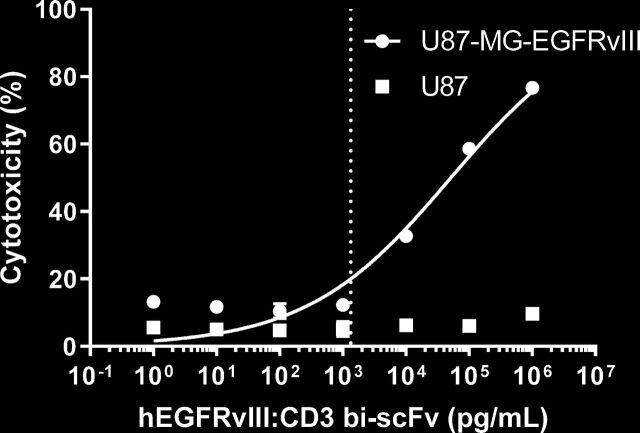
hEGFRvIII:CD3 bi-scFv kills glioma cells in a target and dose dependent manner. Tumor cell cytotoxicity was measured after a 24 hours incubation of hEGFRvIII:CD3 bi-scFv, human PBMCs, and U87-MG-EGFRvIII or U87-MG control cells. The dashed line shows the EC_20_ value for the U87-MG-EGFRvIII group. EC_20_, 20% of the maximal effective concentration; EGFRvIII, epidermal growth factor receptor variant III; PBMCs, peripheral blood mononuclear cells.

Using cGMP-representative hEGFRvIII:CD3 bi-scFv, we repeated the cellular cytotoxicity assay with U87-MG-EGFRvIII and U87-MG control cells (figure 5). As previously shown, tumor cell killing was specific to the EGFRvIII antigen and dose dependent on hEGFRvIII:CD3 bi-scFv. The EC_20_ corresponds to 1.34 ng/mL.

### Dose selection based on an in vitro MABEL approach

As recommended by drug regulatory agencies, we used the EC_20_ of hEGFRvIII:CD3 bi-scFv from all of our in vitro experiments to determine the human starting dose based on a MABEL approach (table 1). In the following sections, we then compare this dose to a HED of our effective in vivo concentration and ensure that the theoretical human CD3 receptor occupancy is below standard values.

**Table 1:** EC_20_ values and corresponding HEDs of the performed in vitro assays

Method	EC_20_ (ng/mL)	Dose (µg)/70 kg patient	Dose (ng)/patient kg
Binding to CD3	150	450	6428
Binding to EGFRvIII	10 307	30 921	441 728
Cytokine release			
IFN-γ	42.3	127	1813
IL-6	12.5	37.5	536
IL-1β	Ambiguous		
MIP-1α	14.7	44.1	630
GM-CSF	27.6	82.8	1183
TNFα	16.5	49.5	707
T cell activation	1021	3063	43 757
T cell proliferation	70.2	210	3008
Tumor cell cytotoxicity	1.34	4.02	57.4

EC_20_, 20% of the maximal effective concentration; EGFRvIII, epidermal growth factor receptor variant III;; HEDs, human equivalent doses.

To calculate the human starting dose, we took the concentration where hEGFRvIII:CD3 bi-scFv showed 20% pharmacologic activity (ie, the EC_20_) for each assay and calculated the amount of drug it would take to reach that concentration in a human, based on an average plasma volume of 3 L and an average patient body weight (BW) of 70 kg (table 1). The MABEL and safe starting dose was thus represented by the most sensitive assay or smallest starting dose.

Our most sensitive assay, based on the lowest EC_20_ concentration, was the tumor cell cytotoxicity assay (figure 5). With an EC_20_ of 1.34 ng/mL, this corresponds to a human starting dose of 4020 ng per patient or 57.4 ng drug/patient kg.

### hEGFRvIII:CD3 bi-scFv extends survival of orthotopic gliomas and is safe in vivo

**Figure 6:**
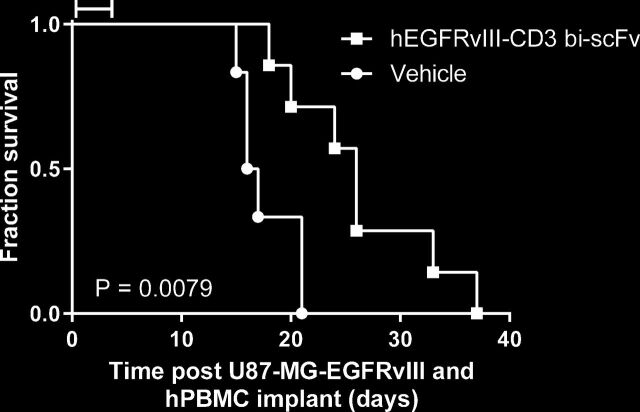
hEGFRvIII:CD3 bi-scFv extends survival in vivo. Immunodeficient NSG mice were coimplanted with U87-MG-EGFRvIII and human PBMCs. Subsequently, mice in the treatment group received 50 µg hEGFRvIII:CD3 bi-scFv daily for 5 days via intravenous tail injection. Bar indicates time of hEGFRvIII:CD3 bi-scFv treatment. EGFRvIII, epidermal growth factor receptor variant III; NSG, NOD-*scid* gamma; PBMCs, peripheral blood mononuclear cells.

We previously showed that hEGFRvIII:CD3 bi-scFv effectively treats patient-derived and murine EGFRvIII-positive malignant glioma in both subcutaneous and orthotopic models.^6^ To show equivalency of cGMP-representative hEGFRvIII:CD3 bi-scFv in vivo, we performed a xenograft experiment in immunodeficient NSG mice (figure 6). Mice were coimplanted orthotopically with human malignant glioma U87-MG-EGFRvIII and human PBMCs. Subsequently, mice in the treatment group were injected intravenously daily for 5 days with 2.5 mg/kg hEGFRvIII:CD3 bi-scFv. Treatment with clinical grade drug significantly extended survival compared with the control group (p=0.0079). Furthermore, a single-dose toxicity study of hhEGFRvIII:CD3 bi-scFv in human CD3 transgenic mice showed that the drug was safe up to at least a dose of 10 mg/kg (maximum tested dose; data unpublished). Overall, based on the data presented here and our other published studies in various glioma and mice models, we see robust treatment efficacy when mice are administered daily doses of either 2.5 or 5 mg/kg hEGFRvIII:CD3 bi-scFv.^6 19^

### The in vivo efficacious and safe dose

Based on this collective data, a daily dose of 2.5 or 5 mg/kg hEGFRvIII:CD3 bi-scFv is efficacious in mouse models. Using the average concentration at steady state (C_ave, ss_) of this dosing regimen in immunocompetent C57BL/6 mice, a human equivalent dose was calculated from the following equation, using predicted human clearance values:

$$Dose=CL_{hu}\times C_{eff,ss}\times Tau,$$

where CL_hu_ is the predicted human clearance, C_eff, ss_ the predicted efficacious concentration at steady state, and Tau the dosing interval.

The predicted human clearance value was based on the clearance of the clinically approved bi-scFv blinatumomab, which has similar architecture and pharmacokinetic properties. Blinatumomab’s clearance is 43 mL/h/kg in adults.^20^ The C_eff, ss_ was assumed to be equal to the average concentration at steady state (C_ave, ss_) based on our pharmacokinetic study using the 5 mg/kg dose in human CD3 transgenic mice (0.583 µg/mL).^11^ A Tau of 24 hours was used.

Using these parameters, a human equivalent dose of 0.6 mg drug/kg of BW was calculated. In comparison, our calculated MABEL dose of 57.4 ng drug/kg of BW is 10,400-fold lower than the murine in vivo efficacious dose, showing a clear safety margin of the MABEL approach.

### Theoretical receptor occupancy is low at our MABEL dose

Theoretical receptor occupancy of target antigens constitutes an important consideration for dose selection. We assessed the theoretical receptor occupancy of hEGFRvIII:CD3 bi-scFv based on our MABEL-based starting dose. As outlined by Lowe *et al*, there are two commonly used algorithms for calculating drug-target occupancy.^21^ Method 1 is based on the Michaelis-Menten equilibrium.

Method 1: $\%RO=\frac{\left[Ab\right]}{\left[Ab\right]+K_{D}}\times 100\%$

where RO is the receptor occupancy, Ab is the concentration of antibody (which corresponds to our MABEL-based starting drug concentration of 1.34 ng/mL (26.3 pM)) and K_D_ is the equilibrium dissociation constant.

However, if there are known to be significant quantities of target receptors in the system, then the Michaelis-Menten equation is no longer valid. In these situations, Method 2, which corrects for the high target expression levels, can be applied.

Method 2:

$$\%RO=\frac{\left(K_{D}+TD+TT\right)-{\left({\left(K_{D}+TD+TT\right)}^{2}-4\times TD\times TT\right)}^{1/2}}{2\times TT}\times 100\%$$

where RO is receptor occupancy, K_D_ is the equilibrium dissociation constant, TD is total drug concentration (which corresponds to our MABEL-based starting drug concentration of 1.34 ng/mL (26.3 pM)), and TT is the total target concentration.

The TT concentration was calculated as follows:

$$TT=\frac{n_{1}*n_{2}}{N_{A}}$$

where n_1_ is the number of CD3 receptors per cell (6.11×10^4^), n_2_ is the number of T cells per volume (1.3×10^9^ L^−1^), and N_A_ is the Avogadro constant.

Using surface plasmon resonance, we previously determined the target binding kinetics of hEGFRvIII:CD3 bi-scFv.^6^ The association rate constant (k_a_), dissociation rate constant (k_d_), and equilibrium dissociation constant (K_D_) for EGFRvIII were 4.45×10^4^ M^−1^s^−1^, 1.24×10^−3^ s^−1^, and 2.78×10^−8^ M, respectively. The association rate constant (k_a_), dissociation rate constant (k_d_), and equilibrium dissociation constant (K_D_) for CD3 epsilon were 5.35×10^4^ M^−1^s^−1^, 8.36×10^−4^ s^−1^, and 1.56×10^−8^ M, respectively. No binding to EGFR wild type was detected. However, since hEGFRvIII:CD3 bi-scFv is a bispecific antibody that requires simultaneous engagement of both target antigens expressed on different cell types (EGFRvIII on tumor cells and CD3 on T cells), one cannot calculate the receptor occupancy of both targets at the same time. Furthermore, since EGFRvIII expression is restricted to tumor cells within the brain (and thus behind the blood-brain barrier), the concentration of hEGFRvIII:CD3 bi-scFv available to bind EGFRvIII is still unknown. Thus, we restricted our receptor occupancy calculations to the ubiquitous CD3 receptor.

According to both methods, the theoretical CD3 receptor occupancy is 0.17% for hEGFRvIII:CD3 bi-scFv based on our MABEL-based starting dose.

## Discussion

Immunotherapies are a diverse category of potent immune-activating molecules. In oncology, recently approved modalities such as bispecific antibodies, checkpoint inhibitors, and engineered T cells have vastly improved the survival and quality of life in patients suffering from a variety of devastating hematologic and solid tumors.^22–24^ Three bispecific antibodies—blinatumomab, catumaxomab, and emicizumab—have received market approval to date.^25^ While catumaxomab was withdrawn from the EU market in 2017, the impressive clinical efficacy of blinatumomab, a CD3 × CD19 engaging T cell redirecting bispecific antibody, has sparked widespread interest in the bispecific platform. In fact, as reported by Labrijn *et al* over 85 bispecific antibodies are currently in clinical development, with over half of them targeting the T cell receptor CD3.^25^

The strong commercial interest in the bispecific platform arises in part from the flexibility to suppress, activate, and redirect cells based on their surface proteins using relatively simple engineered proteins. However, clinical experience has shown that both single and dual specific agonistic antibodies can lead to life-threatening side effects. In 2006, TeGenero initiated a phase I clinical trial in six healthy human volunteers for testing TGN1412.^26^ Targeting CD28, a costimulatory receptor on T cells, TGN1412 is a monoclonal antibody that can activate T cells irrespective of the signal received from the T cell receptors.^26^ Termed a CD28 superagonist, TGN1412 was infused at a starting dose 500 times smaller than that found to be safe in animal studies.^26^ Minutes after the first infusion, all six patients experienced severe adverse reactions, including multiorgan failure, due to the rapid release of cytokines by activated T cells.^26^ Blinatumomab, a bispecific T cell engager for treating CD19 positive B cell malignancies, was approved by the FDA in 2014 for the treatment of relapsed or refractory B-cell precursor acute lymphoblastic leukemia in adults and children.^20^ Across Amgen’s six blinatumomab trials, ranging from phase I to III and involving 712 ALL patients, 25 patients died due to treatment related adverse events (most due to cytokine release syndrome).^27^ As such, blinatumomab’s package insert clearly warns ‘BLINCYTO may cause serious side effects that can be severe, life-threatening, or lead to death, including: … Cytokine Release Syndrome and … Neurologic problems’.^20^

Over the past decade, both the EMA and the FDA released guidance on selecting the maximum recommended starting dose for agonistic single and dual specific antibodies based on the MABEL.^15–17^ The MABEL approach holistically considers mechanistic ex vivo/in vitro studies, preclinical pharmacology and toxicology investigations, in vivo models, and pharmacodynamic modeling for selecting a FIH starting dose.^28^ By integrating all available in vivo and in vitro data, MABEL aims to provide a safe human starting dose.

While MABEL calculations have become standard for CD3 engaging bispecific antibodies and a standard component of an IND application, pharmaceutical companies rarely publish detailed accounts of this crucial translational step. An FDA publication by Saber *et al* highlights and compares the variety of MABEL approaches used by the sponsors.^29^ However, the publication does not disclose specific calculations and states that one sponsor even failed to disclose their calculations to the FDA. In fact, we found only one other detailed study, published by Dudal *et al*, that describes the MABEL approach used to calculate the FIH dose of the Roche bispecific antibody carcinoembryonic antigen T cell bispecific antibody (CEA TCB).^30^

In this study, we aim to provide a detailed account of our FIH dose selection based on the MABEL approach. Our drug, hEGFRvIII:CD3 bi-scFv, is a truncated 50.9 kDa bispecific antibody that binds CD3 on T cells and EGFRvIII, a tumor-specific antigen often expressed in malignant glioma. Our previous works showed that hEGFRvIII:CD3 bi-scFv effectively treats malignant glioma, though it’s small size and lack of a fragment crystallizable region lead to a short half-life in vivo.^6 11 19^ A contract manufacturing organization recently completed the production of cGMP hEGFRvIII:CD3 bi-scFv drug material on our behalf. In order to calculate a FIH starting dose, we conducted and present in this work a series of in vitro and in vivo studies using cGMP-representative hEGFRvIII:CD3 bi-scFv. With these data we (1) calculated a starting dose based on a MABEL approach using our ex vivo/in vitro assays, (2) compared our starting dose to the preclinical in vivo efficacious dose, and (3) modeled the human receptor occupancy we expect at our starting dose.

In order to assess the pharmacologic effect of hEGFRvIII:CD3 bi-scFv, we conducted a myriad of assays that investigated the concentration-dependent effects our molecule has on its target cells. Binding studies showed that our molecule only binds human CD3 receptors (figure 1) and the mutated EGFRvIII—but not the wild type—receptor (figure 2). In our cytokine release, T cell activation, proliferation, and tumor cell lysis assays, hEGFRvIII:CD3 bi-scFv only caused T cell activity in the presence of EGFRvIII expressing tumor cells, highlighting the specificity and potency of our molecule (figure 3–5). We based our MABEL approach on the concentration where hEGFRvIII:CD3 bi-scFv showed 20% of its maximum activity (EC_20_) in the most sensitive assay and converted that concentration into a patient dose (table 1). The most sensitive assay was tumor cell lysis which translates to a starting dose of 57.4 ng hEGFRvIII:CD3 bi-scFv/patient kg.

Based on our in vivo efficacious dose, our in vivo toxicity study, and the predicted human clearance of hEGFRvIII:CD3 bi-scFv, we calculated the predicted effective dose to be 0.6 mg/patient kg. Even when applying a safety factor of 10, as is frequently done in the NOAEL approach, our MABEL dose is still 1040-fold more conservative than had we based our human starting dose calculations solely on our in vivo data.

Target receptor occupancy by antibodies is a crucial determinant of their effect. Complete, that is, 100%, receptor occupancy would therefore result in the maximum expected pharmacodynamics effect. As such, for agonistic antibodies, a safe first-in-human dose should minimize an initial pharmacodynamics effect by ensuring very low receptor occupancy by the antibody (typically <<10%).^31^ In the case of the disastrous CD28 superagonist TGN1412 clinical trial, post-trial analyses revealed that the administered human dose resulted in approximately 90% receptor occupancy.^32 33^ Based on our MABEL starting dose for hEGFRvIII:CD3 bi-scFv we determined a theoretical human receptor occupancy of 0.17%. This receptor occupancy is far below the upper 10% limit and also similar to the first-in-human dose used for Roche’s CEA TCB.^30^

Finally, it is well known that EGFRvIII displays heterogeneous expression within tumors. While this presents a potential limitation of hEGFRvIII:CD3 bi-scFv, we have recently demonstrated that our molecule is capable of eliminating tumors with heterogeneous EGFRvIII expression.^6^ Our studies utilizing patient-derived xenografts with heterogeneous EGFRvIII expression revealed that were are able to effectively treat both subcutaneous and orthotopic xenografts. Furthermore, while EGFRvIII targeting via vaccination or with CAR T cell therapy has shown disappointing results in clinical trials, the degree of tumor penetration and unique mechanism of action of truncated bispecific molecules could prove beneficial in GBM. Therefore, we believe that hEGFRvIII:CD3 bi-scFv may have superior efficacy when compared with vaccines and CAR T cell therapy and could potentially be efficacious in GBM patients with heterogenous EGFRvIII expression.

In conclusion, the comparison of our MABEL-based starting dose to our in vivo efficacious dose and the theoretical human receptor occupancy strongly supports that our maximum recommended starting dose of 57.4 ng hEGFRvIII:CD3 bi-scFv per patient kg will be safe. Combined with our prior work on GBM with heterogeneous EGFRvIII expression, we believe that hEGFRvIII:CD3 bi-scFv is a promising therapeutic modality for treating patients with EGFRvIII positive GBM.
